# Incremental prognostic value of global longitudinal strain in patients with type 2 diabetes mellitus

**DOI:** 10.1186/s12933-016-0333-5

**Published:** 2016-02-03

**Authors:** Ju-Hua Liu, Yan Chen, Michele Yuen, Zhe Zhen, Carmen Wing-Sze Chan, Karen Siu-Ling Lam, Hung-Fat Tse, Kai-Hang Yiu

**Affiliations:** Division of Cardiology, Department of Medicine, The University of Hong Kong, Queen Mary Hospital, Rm 1929b, Block K, Hong Kong, China; Department of Medicine, Meishan People′s Hospital, Meishan, Sichuan China; Division of Endocrinology, Department of Medicine, The University of Hong Kong, Queen Mary Hospital, Hong Kong, China; Research Centre of Heart, Brain, Hormone and Healthy Aging, Li Ka Shing Faculty of Medicine, the University of Hong Kong, Hong Kong, China; Division of Cardiology, Department of Medicine, the University of Hong Kong Shenzhen Hospital, Hong Kong, China

**Keywords:** Type 2 diabetes mellitus, Left ventricular function, Cardiovascular events

## Abstract

**Background:**

Patients with type 2 diabetes mellitus (T2DM) are at high risk for cardiovascular events. The aim of the study was to assess whether global longitudinal strain (GLS) provides prognostic value in these patients.

**Methods:**

A total of 247 T2DM patients without history of cardiovascular complications and participated in the CDATS study were prospectively enrolled. Left ventricular (LV) systolic function was assessed by LV ejection fraction and speckle tracking derived LV systolic GLS. Diastolic function was assessed by E/E′ ratio defined as the passive trans-mitral LV inflow velocity to tissue Doppler imaging velocity of the medial mitral annulus. Cardiovascular event included acute coronary syndrome, cerebrovascular stroke, hospitalization for heart failure and cardiovascular death.

**Results:**

A total of 18 cardiovascular events occurred during a median follow-up duration of 33 months. Both E/E′ ratio [hazard ratio (HR) 1.15, P < 0.01] and GLS (HR 1.39, P < 0.01) were independently associated with cardiovascular events. Importantly, GLS provided incremental prognostic information in addition to clinical data, HbA1c and E/E′ ratio (Chi square 77.46, P = 0.04). Receiver-operator characteristic curve analysis demonstrated that E/E′ ratio [area under curve (AUC) 0.66, P = 0.03] and GLS (AUC 0.72, P < 0.01) were strong predictors of cardiovascular events. Kaplan–Meier analysis showed that patients with E/E′ > 13.6 or GLS > −17.9 % were associated with cardiovascular events. The presence of either a high E/E′ ratio or an impaired GLS provides an excellent negative predictive value of cardiovascular events in these patients.

**Conclusions:**

In T2DM patients with no history of cardiovascular disease, impaired GLS was associated with cardiovascular events and provided incremental prognostic value.

## Background

The number of patients with type 2 diabetes mellitus (T2DM) is increasing rapidly in both developed and developing countries and will reach 380 million people by 2025 [1]. Furthermore, approximately half of the patients with T2DM will die prematurely due to a cardiovascular disease that contributes as a major global cause of early mortality [2]. Therefore accurate risk stratification for these patients is important in order to prevent the occurrence of cardiovascular events.

Prior studies have demonstrated that clinical demographics and HbA1c level can help identify patients with T2DM at high risk for cardiovascular events [3, 4]. In addition, the presence of diastolic dysfunction detected by conventional echocardiography has been shown to help risk stratification in these patients [5, 6]. Recently, the use of advanced analyzing method, namely global longitudinal strain (GLS) analysis derived from 2-dimensional (2D) speckle tracking, enables the detection of subtle systolic dysfunction beyond conventional echocardiography assessment [7, 8]. Whether the presence of impaired systolic strain in patients with T2DM with no previous history of cardiovascular complications predicts future cardiovascular events is however uncertain. The aim of the present study was to assess the incremental prognostic value of systolic GLS to predict for cardiovascular events in T2DM patients with no history of cardiovascular complication in addition to clinical data, HbA1c level and diastolic dysfunction.

## Methods

### Study population

A total of 333 patients with T2DM, as defined by World Health Organization criteria, were recruited at the Queen Mary Hospital, Hong Kong from March 2007 to March 2014. Patients who had a documented history of cardiovascular disease including coronary artery disease (n = 16), paroxysmal or chronic atrial fibrillation (n = 8), cerebrovascular stroke (n = 9), hospitalization for heart failure (n = 7), cardiac valvular disease (n = 5), or refused to participate (n = 11) were excluded. Maximum exercise treadmill testing was performed in the remaining patients at baseline to exclude patients with potential ischemic heart disease (n = 20). Patients had poor echocardiography image quality to analysis were also excluded (n = 10). The final 247 patients were enrolled for the present study. Written informed consent was obtained from all participants. The study was approved by the local institutional review board of the Hong Kong West Cluster and was conducted according to the Declaration of Helsinki. This study is part of the Chinese Diabetic Heart Study (CDATS) to evaluate cardiovascular manifestation in Chinese patients with T2DM, an attempt to evaluate the pathophysiology and potential therapies in these patients [9].

### Study protocol

All patients underwent a complete physical examination and an interview to establish baseline characteristics. Blood pressure was measured after patients were rested for at least 5 min. Smoking status was recorded as positive if patients had smoked (ever or current). BMI was calculated in kg/m^2^. Hypertension was defined as resting systolic or diastolic blood pressure ≥140/90 mmHg at two different clinic visits or prescription of antihypertensive medication. Data on medications were ‘retrieved from patients’ electronic medical record, including anti-hypertensive and anti-diabetic medication. Fasting blood samples were obtained to measure hemoglobinA1c, fasting glucose, total cholesterol, triglyceride, high-density lipoprotein cholesterol, low-density lipoprotein cholesterol and serum creatinine levels.

### Conventional echocardiography

Transthoracic echocardiographic examination was performed in all patients using a commercially available echocardiography system (Vingmed Vivid 7, General Electric Vingmed Ultrasound, Milwaukee, WI, USA) with the patient lying in the lateral decubitus position. A 3.5-MHz transducer was used to obtain images that were digitally stored in cine-loop format. Off-line analysis was performed using the EchoPAC version 108.1.5 (General Electric Vingmed, Horten, Norway). Inter-ventricular septal dimension at end-diastole (IVSd), relative wall thickness (RWT) and left ventricular mass index (LVMI) were assessed according to the current recommendations [10]. Left ventricular ejection fraction (LVEF) was determined from apical four and two-chamber views using the modified Simpson’s biplane method. Evaluation of LV diastolic function was based on the pulsed-wave Doppler of mitral valve inflow. Peak velocity in early diastole (E-wave) and late diastole (A-wave) was measured and the E/A ratio was calculated. Pulsed wave tissue Doppler imaging was used to measure the early diastolic velocity (E′) with the sample volume placed at the septum annulus of mitral valve. In addition, the E/E′ ratio was calculated as an estimation of LV filling pressure [11].

## 2D speckle tracking strain analysis

LV systolic GLS was measured from 3 apical views: 2-chamber view (anterior and inferior walls), 4-chamber view (poster-septum and lateral walls) and 3-chamber view (anterior-septum and posterior wall). Each wall was subsequently divided into 3 areas (basal, mid and apical) and a total of 18 segmental strain curves were obtained. Left ventricular systolic GLS was calculated as the average value of the 3 apical strain peak values at systole.

### Cardiovascular event

Cardiovascular events were defined as acute coronary syndrome (ACS), cerebrovascular stroke, cardiovascular death and hospitalization for heart failure. The definition of ACS was based on the presence of typical chest pain, elevated cardiac enzyme levels, and typical electrocardiogram changes [12]. Hospitalization for heart failure was defined as admission due to dyspnea with chest radiographic evidence of pulmonary congestion and treatment with intravenous diuretics. Cardiovascular death was defined as patients who died following a stroke or heart failure or acute myocardial infarction. If patients had multiple cardiovascular events, only the first event was coded. In the study end points, patients with cardiovascular event were followed until the first episode of cardiovascular events, the others were followed until March 2015 or the date of death.

## Statistical analysis

Data are expressed as mean ± standard deviation for continuous variables and frequencies or proportions for categorical variables. Clinical predictors of cardiovascular event were assessed by univariate Cox regression analysis. Independent echocardiography predictor of cardiovascular event was assessed by univariate and multivariable Cox regression analysis adjusted for age, gender and covariates which are significant variables in the clinical data. A nested Cox proportional hazard regression analysis was used to investigate incremental prognostic value of the predictors. To take into account the time-dependent characteristics of receiver-operating characteristic curves, the prognostic model was assessed with the receiver-operating characteristic curves. The optimal cutoff value was defined as the maximized value for the sum of sensitivity and specificity. The cumulative probability of cardiovascular event during the follow-up period was estimated using the Kaplan–Meier method and compared with the log-rank test. For Inter-observer reproducibility of echocardiographic parameters was assessed by intra-class correlation coefficient; 24 subjects were randomly chosen for this analysis. Statistical analyses were performed using standard statistical computer software SPSS for window (Version 17.0). All P values reported are 2-sided for consistency and P < 0.05 was considered statistically significant.

## Results

### Clinical data and predictors of cardiovascular events

Baseline clinical characteristics of the enrolled patients and predictors of cardiovascular events are shown in Table 1. The mean age was 60 years and there was an even gender distribution. A total of 18 cardiovascular events were recorded during a median follow-up duration of 33 months; 7 patients diagnosed with ACS, 3 patients with hospitalizations for heart failure, 6 patients with cerebrovascular stroke and 2 cardiovascular deaths due to refractory heart failure.

**Table 1:** Clinical data of patients and Cox regression analysis in association with cardiovascular events

Variable	Total (n = 247)	B	HR (95 % CI)	P
Demographic data				
Age, years	59.8 ± 9.5	0.07	1.08 (1.03–1.13)	<*0.01*
Female, n (%)	120 (48.6)	0.77	2.15 (0.80–5.76)	0.13
Body mass index, kg/m^2^	26.2 ± 4.8	−0.08	0.93 (0.82–1.04)	0.20
SBP (mmHg)	136.3 ± 18.7	−0.001	1.00 (0.97–1.03)	0.93
DBP (mmHg)	79.1 ± 8.8	−0.03	0.97 (0.92–1.03)	0.33
Medical history				
Diabetes durations, years	13.5 ± 8.2	0.03	1.03 (0.99–1.08)	0.15
Hypertension, n (%)	186 (75.3)	1.06	2.90 (0.67–12.6)	0.16
Hyperlipidemia, n (%)	130 (52.6)	1.09	2.96 (1.05–8.37)	*0.04*
Smoking, n (%)	55 (22.3)	0.17	1.19 (0.39–3.64)	0.76
Medication				
ACEI/ARB, n (%)	134 (54.3)	0.39	1.48 (0.58–3.78)	0.42
Calcium channel blocker, n (%)	105 (42.5)	0.57	1.76 (0.69–4.47)	0.23
B-blocker, n (%)	55 (22.3)	0.10	1.11 (0.36–3.37)	0.86
Biguanides, n (%)	210 (85.0)	−0.23	0.80 (0.28–2.30)	0.67
Insulin (%)	110 (44.5)	1.09	2.96 (1.12–7.82)	*0.03*
Blood chemistry				
FBG (mmol/L)	8.0 ± 2.5	−0.26	0.77 (0.58–1.02)	0.06
HbA1c (%)	7.8 ± 1.4	−0.30	0.74 (0.49–1.13)	0.16
TG (mmol/L)	1.4 ± 1.0	−0.24	0.79 (0.43–1.45)	0.44
TC (mmol/L)	4.5 ± 0.8	−0.16	0.86 (0.50–1.48)	0.58
HDL (mmol/L)	1.3 ± 0.4	0.81	2.25 (0.79–6.37)	0.13
LDL (mmol/L)	2.5 ± 0.7	−0.49	0.61 (0.29–1.30)	0.20
Creatinine (mmol/L)	80.2 ± 39.2	0.01	1.01 (1.00–1.01)	*<0.01*

Italic values indicate statistically significant with P < 0.05

*ACEI* angiotensin-converting enzyme inhibitor, *ARB* angiotensin II receptor antagonist, *CI* confidence interval, *DBP* diastolic blood pressure, *FBG* fasting blood glucose, *HbA1c* hemoglobinA1c, *HDL* high-density lipoprotein cholesterol, *HR* hazard ratio, *LDL* low-density lipoprotein cholesterol, *SBP* systolic blood pressure, *TC* total cholesterol, *TG* triglyceride

Clinical predictors of cardiovascular events assessed by univariate Cox regression analysis were age [hazards ratio (HR) 1.08; 95 % confidence interval (CI) 1.03–1.13, P < 0.01], history of hyperlipidemia (HR 2.96, 95 % CI 1.0–8.37, P = 0.04), treatment with insulin (HR 2.96, 95 % CI 1.12–7.82, P = 0.03) and serum creatinine level (HR 1.01, 95 % CI 1.00–1.01, P < 0.01).

### Echocardiography parameters

**Fig. 1:**
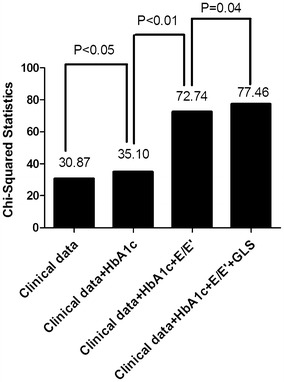
Change in model Chi square with addition of HbA1c, E/E′ ratio and GLS to clinical data (including age, gender, hyperlipidemia, plasma creatinine and treatment with insulin). *E/E′ ratio* the ratio of early peak mitral inflow velocity to early myocardial velocity of mitral annulus at diastole, *GLS* global longitudinal stain, *HbA1c* hemoglobinA1c

All patients had a preserved LVEF (all LVEF >50 %) at baseline and the mean LVEF were 63.2 ± 4.5 %. Echocardiography parameters associated with cardiovascular events were shown in Table 2. Both E/E′ ratio and GLS were significant associated with cardiovascular events in univariate Cox regression analysis. After adjusted Cox regression analysis, E/E′ ratio and GLS remained associated with cardiovascular events. No such association was observed in LVEF and E/A ratio, as well as parameters of LV geometry, including IVSd, RWT and LVMI. To examine the incremental prognostic value of the predictors, we performed a nested Cox proportional hazard regression analysis. A model based on clinical data including age, gender, hyperlipidemia, plasma creatinine and treatment with insulin was improved by HbA1c and E/E′ ratio. The addition of GLS to the model provided incremental prognostic information beyond clinical data, HbA1c and E/E′ ratio (Fig. 1).

**Table 2:** Adjusted Cox regression analysis of individual echocardiography parameters for cardiovascular events

Variable	Value (n = 247)	B	HR (95 % CI)	P
IVSd (cm)	1.13 ± 0.19			
Univariate analysis		2.01	7.43 (0.66–83.7)	0.10
Adjusted clinical data^a^		1.43	4.17 (0.31–56.8)	0.28
Adjusted clinical data + HbA1c		1.87	6.50 (0.47–89.5)	0.16
RWT, ratio	0.50 ± 0.09			
Univariate analysis		−0.47	0.63 (0.002–171.5)	0.87
Adjusted clinical data^a^		−1.25	0.29 (0.001–89.3)	0.67
Adjusted clinical data + HbA1c		0.87	2.39 (0.01–783.1)	0.77
LVMI (g/m^2)^	100.7 ± 24.7			
Univariate analysis		0.02	1.02 (1.00–1.03)	0.07
Adjusted clinical data^a^		0.01	1.01 (0.99–1.04)	0.29
Adjusted clinical data + HbA1c		0.01	1.01 (0.99–1.04)	0.27
LVEF (%)	63.2 ± 4.5			
Univariate analysis		−0.08	0.92 (0.83–1.02)	0.13
Adjusted clinical data^a^		−0.07	0.94 (0.85–1.04)	0.19
Adjusted clinical data + HbA1c		−0.08	0.93 (0.84–1.02)	0.11
E/A ratio	0.94 ± 0.31			
Univariate analysis		−2.51	0.08 (0.01–0.98)	0.05
Adjusted clinical data^a^		−1.05	0.35 (0.02–6.09)	0.47
Adjusted clinical data + HbA1c		−1.62	0.20 (0.01–4.04)	0.29
E/E′ ratio	10.4 ± 4.5			
Univariate analysis		0.15	1.16 (1.10–1.22)	*<0.01*
Adjusted clinical data^a^		0.14	1.16 (1.09–1.23)	*<0.01*
Adjusted clinical data + HbA1c		0.14	1.15 (1.08–1.22)	*<0.01*
GLS (%)	−18.1 ± 2.4			
Univariate analysis		0.27	1.31 (1.10–1.56)	*<0.01*
Adjusted clinical data^a^		0.29	1.35 (1.10–1.65)	*<0.01*
Adjusted clinical data + HbA1c		0.33	1.39 (1.14–1.70)	*<0.01*

Italic values indicate statistically significant with P < 0.05

Abbreviations: Similar to Table 1, *E/A ratio* the ratio of peak mitral flow velocity in early and late at diastole, *E/E′* *ratio* the ratio of early peak mitral inflow velocity to early myocardial velocity of mitral annulus at diastole, *GLS* global longitudinal stain, *IVSd* Inter-ventricular septal dimension at end-diastole, *LVEF* left ventricular ejection fraction, *LVMI* left ventricular mass index, *RWT* relative wall thickness

^a^Clinical data includes age, gender, hyperlipidemia, plasma creatinine and treatment with insulin

### E/E′ ratio and GLS are associated with cardiovascular events

**Fig. 2:**
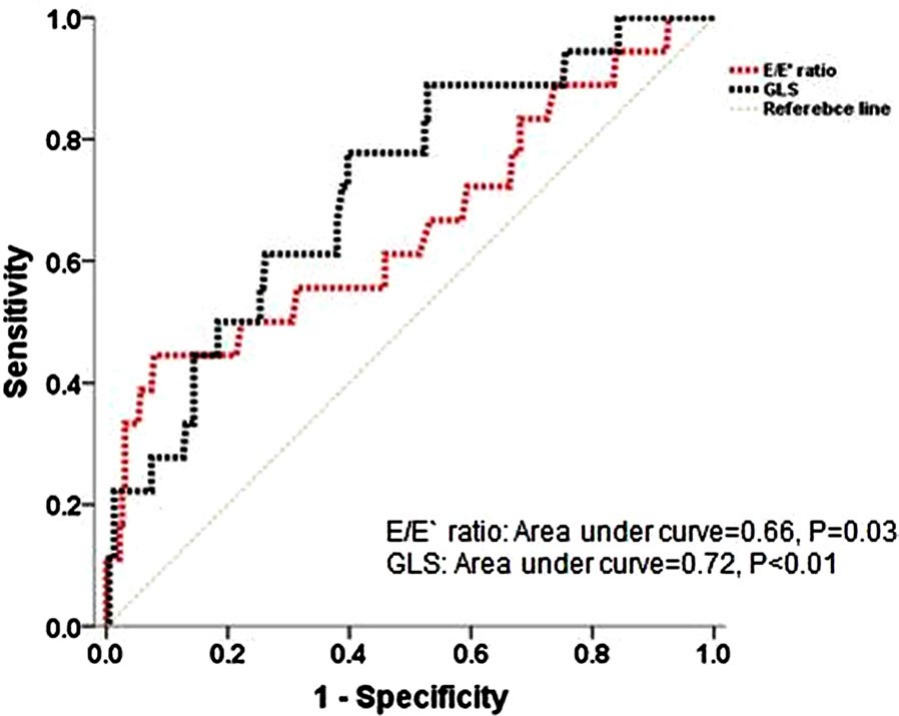
Receiver-operating characteristic curves for prediction of cardiovascular events in patients with type 2 diabetes mellitus using E/E′ ratio and GLS. Abbreviations: Similar to Fig. 1

**Fig. 3:**
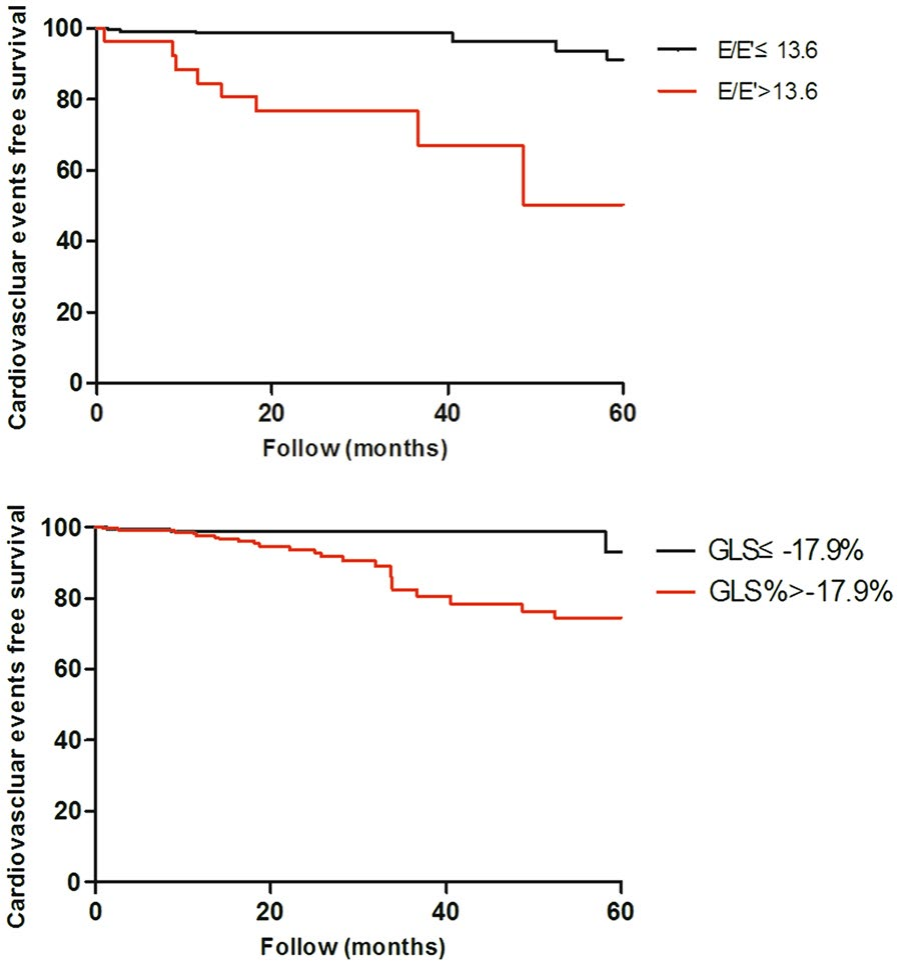
Kaplan–Meier analysis of cardiovascular event-free survival according to GLS > −17.9 % compared with GLS ≤ −17.9 % (Log rank χ^2^ = 6.52 P = 0.01), and E/E > 13.6 compared with E/E′ ≤ 13.6 (Log rank χ^2^ = 26.73 P < 0.01). Abbreviations: Similar to Fig. 1

The receiver-operating characteristic curve was generated to determine the accuracy of E/E′ ratio and GLS in association with cardiovascular events. As shown in Fig. 2, E/E′ ratio and GLS were significantly associated with cardiovascular events. Kaplan–Meier analysis demonstrated that E/E′ > 13.6 or GLS > −17.9 % was associated with cardiovascular events (Fig. 3). In the present study, a total of 10.5 % (n = 26) patients had a E/E′ > 13.6 and 42.5 % (n = 105) patients had a GLS > −17.9 %. A cut-off value of 13.6 for E/E′ ratio provided a sensitivity of 44.4 % and specificity of 92.1 %, whereas a cutoff value of −17.9 % for GLS showed a sensitivity of 77.8 % and specificity of 60.3 %. A combination of >13.6 E/E′ and > −17.9 % GLS had a sensitivity of 38.9 %, a specificity of 97.4 %, a positive predictive value of 53.8 %, and a negative predictive value of 95.3 %. Importantly, the presence of either >13.6 E/E′ or > −17.9 % GLS had a sensitivity of 83.3 %, a specificity of 55.0 %, a positive predictive value of 12.7 %, and a negative predictive value of 97.7 %.

### Reproducibility of E/E′ and GLS

Variability was measured for left ventricular E/E′ and GLS. Intra-class correlation coefficient showed good inter-observer agreement with non-significant differences. Intra-observer coefficient was 0.91 for E/E′ and 0.99 for GLS.

## Discussion

The present study demonstrates that GLS measured by 2D speckle tracking derived strain is independently associated with cardiovascular events in T2DM patients with no history of cardiovascular complications. Importantly, assessment of GLS provides incremental prognostic value to clinical data, HbA1c level and diastolic function assessed by E/E′ ratio in those patients.

### Association between LV diastolic dysfunction and cardiovascular events

To better risk stratify patients with T2DM, prior studies have developed scoring systems based on HbA1c levels and clinical demographics [13–15]. Results have shown that patients with severe disease status were more frequent to develop cardiovascular events. Similarly, the present study demonstrated that age, medical history of hyperlipidemia, treatment with insulin and serum creatinine levels were significantly associated with cardiovascular events. Nonetheless, these scoring systems provide modest prognostic value and tend to overestimate the overall risk [16–18]. As a result, additional methods to improve risk stratification in these patients are required. Echocardiography is a non-invasive modality to assess cardiac function. Diastolic dysfunction, a common finding in patients with T2DM that can be readily assessed by echocardiography [19], is closely associated with the state of insulin resistance [20], impaired fasting glucose [21] and medical history of coronary microvascular disease [22]. In particular, an impaired diastolic function defined by an E/E′ ratio of >15 has been shown to be associated with 1.08–1.61 fold increased risk of cardiovascular events [5]. In patients with T2DM with no history of cardiovascular complications, E/E′ ratio tends only to be mildly elevated [7, 23]. Accordingly, only a small fraction of these patients will reach an E/E′ ratio of >15. In the present study, the E/E′ ratio was only mildly elevated and only 14 (5.7 %) patients had an E/E′ ratio of >15. Nonetheless, E/E′ ratio remains significantly associated with cardiovascular events in the present cohort of patients with no history of cardiovascular complications. Further, ROC demonstrated that a cut-off value of 13.6 for E/E′ provides a reasonable sensitivity to predict cardiovascular events. This finding thus supports the assessment of diastolic dysfunction to improve risk stratification, even in patients who have not developed cardiovascular complication.

### Association between LV systolic dysfunction and cardiovascular events

Strain analysis derived from speckle tracking enables detection of subclinical myocardial systolic dysfunction beyond conventional LVEF assessment. Studies have demonstrated that impaired GLS was observed not only in T2DM patients with no history of cardiovascular complications but also in asymptomatic T2DM patients even with normotensive [7, 23, 24], whist coexistent of diabetic complications, hypertension, hypertriglyceridemia and overweight or obesity resulted in further damage to myocardial contractility [25–27]. Nonetheless, limited studies have evaluated the prognostic value of GLS in patients with T2DM. In a study involving 406 patients, GLS did not demonstrate independent prognostic value [5]. While in another study that included only patients who had no history of cardiovascular complication, GLS was able to predict cardiovascular events, a finding confirmed by the present study [23]. Importantly, the current study is the first to demonstrate the incremental prognostic value of GLS beyond clinical data, HbA1c and diastolic function. In particular, a GLS of > −17.9 % provides a sensitivity of 77.8 %. This finding thus highlights the usefulness of GLS assessment in patients with T2DM.

### Clinical implication

The current recommendation of cardiovascular screening for diabetic patients does not include the routine use of echocardiography to detect diastolic or systolic dysfunction [28]. Indeed, both diastolic and systolic dysfunction is common in patients with T2DM, even before they developed cardiovascular complication [7, 29]. The present study demonstrates that the use of echocardiography assessment, particularly diastolic function and systolic strain analysis, provides incremental prognostic value to routine clinical parameters. Importantly, the presence of either E/E′ > 13.6 or GLS > −17.9 % has a high negative predictive value (97.7 %) and may identify patients who are at low risk of cardiovascular events. The present finding thus supports the value of echocardiography assessment to identify low risk patients, before the development of cardiovascular complication and warrants consideration to be performed routinely. Nonetheless, it is important to note that the low specificity of either of these values suggests the limited role to detect high-risk patients. Clinician should therefore consider additional parameters, in addition to E/E′ and GLS, to accurately detect those who are at high-risk for future cardiovascular events. It is worthy of note that the AUC under the ROC of E/E′ and GLS was 66 and 72 %, respectively. Although significant, these parameters only provide moderate discriminating capacity and the overall risk stratification in patients with T2DM should combine with other risk stratification parameters. Furthermore, a total of 13 patients with both E/E′ > 13.6 and GLS > −17.9 %, 2 (15.4 %) patients developed IHD, 2 (15.4 %) patients developed HF and 2 (15.4 %) patients died due to refractory heart failure during follow-up. The potential value of cardiac computed tomography or coronary angiography assessment to detect significant coronary artery disease in these patients would nonetheless require further study.

### Limitation

The current study did not perform coronary artery imaging in all patients to rule out silent ischemia or asymptomatic coronary artery disease. Nonetheless, patients in the present study had no history of myocardial infarction as supported by preserved LVEF and negative stress test in all patients. Indeed, we believe that the present study population may better represent patients that are encountered in clinical setting where coronary artery assessment are not routinely performed according to the current recommendation [28]. Along the same line, the current cohort did not include patients who had already developed cardiovascular complications and the present findings thus need verification for this group of patients by future studies. Furthermore, data on left atrial geometry and function was not included. A larger study should be performed not only to better define the cut-off value of both diastolic and systolic function but also to assess the prognostic value of left atrial size and function. Finally, potential limitations of echocardiography assessment are dependent on high quality of images and appropriate imaging settings, which can be influenced by medical history of morbidity or pulmonary disease.

## Conclusions

The present study demonstrates that impaired GLS was associated with cardiovascular events in T2DM patients with no history of cardiovascular complications. Importantly, GLS provides incremental prognostic value to clinical demographics, HbA1c and diastolic function in these patients. Further, an E/E′ > 13.6 or GLS > −17.9 % provided an excellent negative predictive value for cardiovascular events. Addressing these parameters by echocardiography may improve risk stratification in patients with T2DM with no history of cardiovascular complications.

## Abbreviations

A-wave: peak mitral inflow velocity in late at diastole
E-wave: peak mitral inflow velocity in early at diastole
E′: the early myocardial velocity of medial mitral annulus at diastole
GLS: global longitudinal stain
LVEF: left ventricular ejection fraction
T2DM: type 2 diabetes mellitus
